# Expression of neural cell adhesion molecule L1 (CD171) in neuroectodermal and other tumors. An immunohistochemical study of 5155 tumors and critical evaluation of CD171 prognostic value in gastrointestinal stromal tumors

**DOI:** 10.18632/oncotarget.10527

**Published:** 2016-07-11

**Authors:** Shingo Inaguma, Zengfeng Wang, Jerzy P. Lasota, Markku M. Miettinen

**Affiliations:** ^1^ Laboratory of Pathology, National Cancer Institute, Bethesda, MD, USA; ^2^ Department of Pathology, Aichi Medical University School of Medicine, Nagakute, Japan

**Keywords:** CD171 (NCAM-L1, L1CAM), immunohistochemistry, neuroectodermal tumor, gastrointestinal stromal tumor, mismatch repair deficiency

## Abstract

The neural cell adhesion molecule L1 (CD171) is a multidomain type 1 membrane glycoprotein of the immunoglobulin superfamily important in the nervous system development, kidney morphogenesis, and maintenance of the immune system. Recent studies reported CD171 expression being associated with adverse clinical outcome in different types of cancer and there has been a growing interest in targeting this cell membrane molecule on neoplastic cells by chimeric antigen receptor redirected T lymphocytes or specific antibodies. Nevertheless, conflicting results regarding the prognostic value of CD171 expression in renal cell carcinomas and gastrointestinal stromal tumors were published. In this study, CD171 expression was immunohistochemically analyzed in 5155 epithelial, mesenchymal, melanocytic, and lymphohematopoietic tumors to assess its utility in diagnostic pathology and to pinpoint potential targets for CD171-targeting therapy. A newly developed anti-CD171 rabbit monoclonal antibody, clone 014, was selected from the panel of commercially available CD171 antibodies. Immunohistochemistry was performed using Leica Bond Max automation and multitumor blocks containing up to 60 tumor samples. CD171 was constitutively and strongly expressed in neuroectodermal tumors such as schwannoma, neuroblastoma, and paraganglioma, whereas other mesenchymal tumors including schwannoma mimics showed only rarely CD171 positivity. Frequent CD171-expression was also detected in ovarian serous carcinoma, malignant mesothelioma, and testicular embryonal carcinoma. CD171 immunohistochemistry may have some role in immunophenotypic differential diagnosis of neurogenic tumors and pinpointing potential candidates for anti-CD171 therapy. Though, because of its rare expression and lack of predictive value, CD171 is neither a diagnostic nor prognostic marker for gastrointestinal stromal tumors.

## INTRODUCTION

The neural cell adhesion molecule L1 (CD171, NCAM-L1, L1CAM) was first identified in the central nervous system (CNS) of the mouse. [1] CD171 is a multidomain type 1 transmembrane glycoprotein of the immunoglobulin superfamily composed of six Ig-like domains, five fibronectin type III repeats, transmembrane region, and a highly conserved cytoplasmic tail. [2] CD171 is the member of the L1-family of closely related neural cell adhesion molecules (CAMs). [3] CD171 can bind to itself (homophilic) or heterophilically to other molecules including integrins, CD24, neurocan, neuropilin-1, and other members of the L1 family for signal transduction. [4, 5] The cytoplasmic tail of CD171 can interact with other cytoplasmic proteins such as ankyrin, actin, spectrin, and ezrin-radixin-mesin (ERM) proteins. [6] It is also reported that CD171 is enzymatically cleaved by a disintegrin and metalloproteinase (ADAM) and extra cellular domains were detectable as a soluble form in the blood. [7–9]

CD171 plays an important role in neural development for myelination, fasciculation, axon guidance, and migration of granule cells in cerebellar cortex. [10–12] Consistent with an important role of CD171 in CNS development, CD171 mutations lead to variable abnormalities including mental retardation and anomaly of CNS, referred to as CRASH (corpus callosum hypoplasia, retardation, adducted thumbs, spastic paraplegia and hydrocephalus) syndrome. [5, 13] Outside the nervous system, CD171 appears to be important for kidney morphogenesis [14] and interactions between leukocytes and endothelial cells. [15]

Recently, several studies showed that overexpression of CD171 in ovarian, endometrial, colorectal and non-small cell lung carcinomas correlates with worse clinical outcome. [16–19] In addition, there has been growing interest in targeting this cell membrane molecule on neoplastic cells by chimeric antigen receptor redirected T lymphocytes or specific antibodies. [20–25] However, immunohistochemical data on CD171 expression in germ cell, rare gastrointesitinal and peripheral mesenchymal tumors as well as lymphohematopoietic tumors remains incomplete. Moreover, conflicting data regarding CD171 expression and its prognostic value were reported in renal cell cancers (RCCs) [26–28] and gastrointestinal stromal tumors (GISTs). [9, 29, 30]

The aim of this study was to evaluate potential utility of CD171 immunohistochemistry in diagnostic pathology and to identify additional tumor types for future CD171-targeting therapy.

## RESULTS

### Comparison of three different CD171 antibodies in normal and selected tumor tissues

In normal tissues, all 3 antibodies showed limited CD171 immunoreactivity in peripheral nerves, brain tissue, vascular endothelial cells, dendritic reticulum cells, and renal collecting ducts (Supplementary Figure S1A-S1F). Organs such as adrenocortical glands, liver, pancreas, placenta and thyroid glands were negative except for the peripheral nervous tissue and endothelial cells. Normal squamous, glandular, and parenchymal epithelia of lung, gastrointestinal-, and genitourinary-tracts, and lymphoid cells and histiocytes were also negative. Mouse monoclonal antibodies L1-14.10 and UJ127.11 showed higher background signals in smooth muscle layer of the gastrointestinal-tract and thyroid glands (Supplementary Figure S1A-S1C and S1G-S1I).

Analysis of 240 tumor tissues including clear renal cell carcinoma, schwannoma, gastric conventional GIST, pancreatic neuroendocrine tumor, and paraganglioma, showed comparable staining results with rabbit 014 and mouse L1-14.10 monoclonal antibodies, while UJ127.11 mouse monoclonal antibody revealed the lowest positivity especially in neuroendocrine tumors. However, 3 clear cell renal cell carcinomas, a schwannoma, and a gastric GIST displayed stronger staining with rabbit 014 than with mouse L1-14.10 clone. Also, rabbit monoclonal antibody had the best sensitivity and lack background signal generated by both, L1-14.10 and UJ127.11 antibodies. Sporadically, clone L1-14.10 revealed slightly stronger signal in the normal peripheral nerve bundles contained in neoplastic tissue sample. All these observations are comparable results illustrated in supplementary data (Figure S2A-S2I and Supplementary Table S1). Based on the results of this preliminary study, clone 014 was identified to be the most appropriate antibody for tumor CD171 immunohistochemistry.

### Neurogenic, neuroendocrine and melanocytic tumors

**Figure 1:**
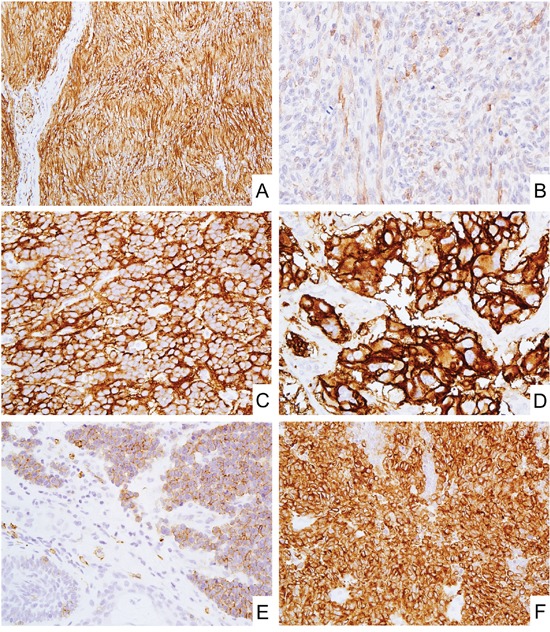
CD171 expression in neural, neuroectodermal and neuroendocrine tumors **A.** Schwannoma showed diffuse and strong CD171 expression. **B.** Malignant peripheral nerve sheath tumor showed weak CD171 expression. **C** and **D.** Blastema component of neuroblastoma (C) and paraganglioma (D) diffusely expressed CD171. **E** and **F.** Merkel cell carcinoma (E) and small cell carcinoma of lung (F) also showed CD171 expression.

The results of CD171 immunostaining of neurogenic, neuroendocrine and melanocytic tumors have been summarized in Table 1. Peripheral and gastrointestinal schwannomas (Figure 1A) showed equally strong positivity in the neoplastic cell populations in nearly all cases. Neurofibromas contained varying numbers of positive Schwann cells in 74% of cases. However, malignant peripheral nerve sheath tumor (MPNST) showed rarer and weaker CD171 expression (32%, Figure 1B).

**Table 1:** CD171 Expression in 749 Neurogenic and Melanocytic Tumors

Tumor Type	Positive	/	All	(%)
Non-GI-tract tumors				
Schwannoma	74	/	74	100.0
Neuroblastoma	29	/	29	100.0
Paraganglioma	65	/	67	97.0
Merkel cell carcinoma	23	/	27	85.2
Pancreas, neuroendocrine tumor	35	/	42	83.3
Lung, small cell carcinoma	32	/	39	82.1
Olfactory neuroblastoma	6	/	8	75.0
Neurofibroma	28	/	38	73.7
Skin, malignant melanoma	57	/	88	64.8
Brain, glioblastoma	26	/	44	59.1
Malignant peripheral nerve sheath tumor	18	/	57	31.6
Nasal Cavity, malignant melanoma	20	/	65	30.8
Vulva and vagina, malignant melanoma	8	/	27	29.6
GI-tract tumors				
Schwannoma, GI-tract	36	/	38	94.7
Primary malignant melanoma, GI-tract	28	/	42	66.7
Metastatic malignant melanoma, GI-tract	37	/	64	57.8

GI: gastrointestinal.

Neuroblastoma and paraganglioma (adrenal and extra-adrenal) were nearly uniformly positive showing strong cytoplasmic and membranous labeling (Figure 1C and 1D).

Neuroendocrine tumors such as merkel cell carcinoma (Figure 1E), small cell carcinoma of lung (Figure 1F), and pancreatic neuroendocrine tumors showed CD171-positivity in 82-85% of cases, generally in a majority of tumor cells.

Cutaneous melanoma (65%), primary (67%) and metastatic (58%) melanomas of gastrointestinal-tract were more often positive than primary sinonasal melanomas (31%) or melanomas of vulva and vagina (30%, Table 1).

### Mesenchymal tumors

**Figure 2:**
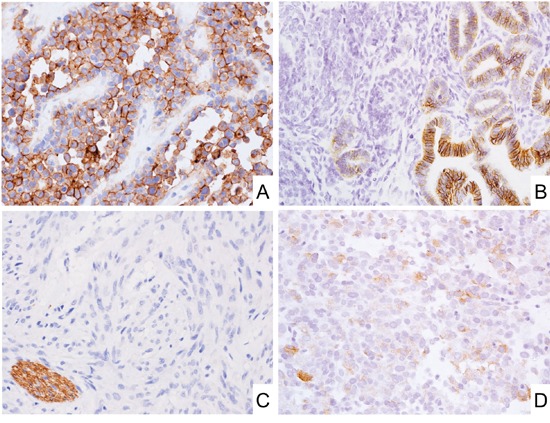
CD171 expression in mesenchymal tumors **A.** Alveolar rhabdomyosarcoma expressed CD171. **B.** Nephroblastoma showed membranous CD171 expression in epithelial components. **C.** Almost all GISTs were negative for CD171. Note that peripheral nerve bundles beside GIST tumor foci were positive for CD171. **D.** SDH-deficient GIST showed weak CD171 expression.

CD171 expressions in mesenchymal tumors have been summarized in Table 2. Of non-gastrointestinal mesenchymal tumors, alveolar rhabdomyosarcoma (50%, Figure 2A), nephroblastoma (38%, Figure 2B), angiosarcoma (36%), desmoplastic small round cell tumors (29%), alveolar soft part sarcoma (18%), PEComa (17%), and embryonal rhabdomyosarcoma (15%), showed CD171 positivity in >10% of cases. In nephroblastoma, the expression was limited to membranes of epithelia differentiating into metanephric tubules and blastema was negative (Figure 2B). Potential schwannoma mimics such as benign fibrous histiocytoma, perineurioma, and solitary fibrous tumors were all negative for CD171.

**Table 2:** CD171 Expression in 1872 Mesenchymal Tumors

Tumor Type	Positive	/	All	(%)
Non-GI-tract tumors				
Alveolar rhabdomyosarcoma	21	/	42	50.0
Nephroblastoma	9	/	24	37.5
Angiosarcoma	35	/	97	36.1
Desmoplastic small round cell tumor	4	/	14	28.6
Alveolar soft part sarcoma	2	/	11	18.2
PEComa	14	/	85	16.5
Embryonal rhabdomyosarcoma	8	/	55	14.5
Synovial sarcoma	3	/	34	8.8
Chordoma	2	/	39	5.1
Benign fibrous histiocytoma	0	/	94	0.0
Kaposi's sarcoma	0	/	35	0.0
Perineurioma	0	/	8	0.0
Solitary fibrous tumor	0	/	28	0.0
GI-tract tumors				
GIST (total)	20	/	1300	1.5
Esophagus	0	/	13	0.0
Stomach (subtotal)	15	/	804	1.9
Conventional GIST	12	/	750	1.6
SDH-defficient GIST	3	/	54	5.6
Duodenum	1	/	53	1.9
Small intestine	0	/	280	0.0
Colorectum	4	/	150	2.7
Fibromyxoma, stomach	2	/	6	33.3
Sarcomas, GI-tract	10	/	48	20.8
Leiomyosarcoma, GI-tract	5	/	29	17.2
Leiomyoma, GI-tract	3	/	45	6.7
Inflammatory fibroid polyp, small intestine	1	/	20	5.0
Clear cell sarcoma, GI-tract	0	/	6	0.0
Glomus tumor, GI-tract	0	/	11	0.0
Inflammatory myofibroblastic tumor, stomach	0	/	6	0.0

GI: gastrointestinal.

Gastrointestinal stromal tumors of various sites only rarely contained CD171-positive tumor cells (1.5%, Figure 2C and Table 2). Slightly higher frequency of positivity was detected in succinate dehydrogenase (SDH)-deficient gastric GISTs (6%, Figure 2D). The majority of positive GISTs showed only weak staining, and moderate labeling was detected in 3 and strong in 2 cases (Table 3). Although 6 of gastric GISTs could be characterized as high-risk tumors, most patients (11/13) were either alive without disease at follow-up or died of other causes (Table 3). No significant prognostic difference was detected between CD171-positive and -negative GIST patients (Supplementary Figure S3). Type and frequency of *KIT* and *PDGFRA* mutations in CD171-positive GISTs mirrored the one seen in randomly selected cohorts of GIST (Table 3). We would like to note that two other mouse monoclonal antibodies, those often used in previous studies, yielded similar negative results in a sub cohort of 57 GISTs studied with all 3 antibodies, however, internal positive control (peripheral nerves) were detected in most cases (Supplementary Table S1 and representative photos in Supplementary Figure S2G-S2I).

**Table 3:** Clinical Data of CD171 Positive GIST Cases

Age	Sex	Primary Site	CD171 Expression		Genetic	Tumor Size	Mitosis	Risk	Follow-up Data	
			Positivity (%)	Intensity	Profile	(cm)	(/50HPF)	Group	(month)	Outcome
41	F	Stomach	100	Moderate	SDH-deficient	7.0	19	6a	180	AWM
20	F	Stomach	100	Weak	SDH-deficient	ND	7	-	ND	
19	M	Stomach	100	Weak	SDH-deficient	4.5	12	5	132	ANR
70	F	Stomach	100	Weak	KIT-Mutant Del 560	3.0	1	2	163	DOC
65	F	Stomach	100	Weak	KIT-Mutant V559D	5.0	0	2	ND	
55	M	Stomach	100	Weak	KIT-Mutant Del 557-558	4.5	6	5	239	DOC
26	M	Stomach	100	Weak	KIT-Mutant Delins 563-576I	5.0	3	2	60	ANR
50	M	Stomach	100	Weak	KIT/PDGFRA Wild Type	5.0	6	5	32	ANR
70	M	Stomach	100	Weak	ND	7.5	0	3a	152	ANR
66	M	Stomach	100	Strong	ND	7.0	>100	6a	5	DOD
62	F	Stomach	90	Weak	PDGFRA-Mutant Delins 842-846E	5.0	11	5	28	DOC
67	M	Stomach	80	Weak	KIT/PDGFRA Wild Type	4.5	0	2	116	ANR
32	M	Stomach	80	Weak	ND	12.0	3	3b	158	DOD
53	M	Stomach	40	Moderate	ND	2.3	2	2	307	ANR
42	M	Stomach	10	Weak	KIT-Mutant Delins 567-576 V	6.0	1	3a	201	ANR
67	M	Duodenum	100	Weak	ND	3.0	>100	-	22	DOD
81	F	Cecum	20	Weak	ND	ND	10	-	ND	
32	M	Colon	10	Weak	KIT/PDGFRA Wild Type	6.0	2	-	ND	
62	M	Colon	60	Strong	ND	7.5	>100	-	ND	
58	M	Rectum	80	Moderate	ND	0.8	44	-	203	DUC

GIST: gastrointestinal stromal tumor, ANR: alive with no recurrence, AWM: alive with metastasis, DOC: died of other cause, DOD: died of disease, DUC: died of unknown cause, ND: no data.

Also, some CD171 positivity was observed in plexiform fibromyxomas (33%), leiomyosarcomas (17%) and non-GIST sarcomas (21%, most commonly dedifferentiated liposarcomas). Other gastrointestinal mesenchymal tumors including clear cell sarcoma were negative with a few exceptions.

### Epithelial neoplasms

**Figure 3:**
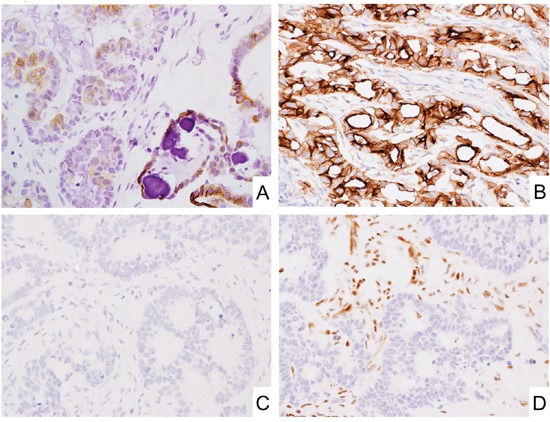
CD171 expression in epithelial tumors **A.** Subpopulation of ovary serous carcinoma showed CD171 expression. **B.** Malignant mesothelioma showed CD171 expression. **C** and **D.** CD171 was expressed at under-detectable level in mismatch repair deficient colorectal adenocarcinoma. Immunostaining for CD171 (C) and MLH1 (D).

CD171 expressions of epithelial tumors have been summarized in Table 4. High frequencies of CD171-positivity were also detected in ovary serous carcinoma (87%, median value of positive cells 60%, Figure 3A), malignant mesothelioma (70%, Figure 3B), and testicular embryonal carcinoma (60%).

**Table 4:** CD171 Expression in 2224 Epithelial Tumors

Tumor Type	Positive	/	All	(%)
Ovary, serous carcinoma	84	/	97	86.6
Malignant mesothelioma	128	/	183	69.9
Testis, embryonal carcinoma	21	/	35	60.0
Colorectum, adenocarcinoma	118	/	210	56.2
Gingiva, squamous cell carcinoma	20	/	43	46.5
Tongue, squamous cell carcinoma	18	/	39	46.2
Uterus, endometrial cancer	32	/	97	33.0
Stomach, adenocarcinoma	31	/	101	30.7
Liver, cholangiocellular carcinoma	5	/	20	25.0
Renal cell carcinoma/ oncocytoma	71	/	333	21.3
Breast, ductal carcinoma	47	/	231	20.3
Urinary tract, urothelial Carcinoma	17	/	92	18.5
Lung, squamous cell carcinoma	8	/	53	15.1
Testis, seminoma	10	/	71	14.1
Adrenocortical gland, adrenocortical cancer	4	/	31	12.9
Tonsil, squamous cell carcinoma	5	/	41	12.2
Lung, adenocarcinoma	4	/	52	7.7
Salivery gland, pleomprphic adenoma	7	/	114	6.1
Thyroid gland, papillary carcinoma	3	/	58	5.2
Thymus, thymoma	3	/	62	4.8
Prostate, adenocarcinoma	2	/	108	1.9
Liver, hepatocellular carcinoma	1	/	92	1.1
Testis, yolk sac tumor	0	/	3	0.0
Breast, lobular carcinoma	0	/	58	0.0

In gastrointestinal adenocarcinomas, colorectal and gastric adenocarcinomas showed 56% and 32% of positivity for CD171, respectively. 11% (23/210) of colorectal adenocarcinomas showed mismatch repair (MMR)-deficient phenotypes. Significantly negative correlation was detected between MMR-deficiency and CD171-expression in colorectal adenocarcinomas (Figure 3C and 3D, and Table 5). On the other hand, 5% (5/92) and 10% (10/100) of gastric adenocarcinomas showed MMR-deficiency and *EBER*-positivity, respectively. Though, no significant correlations were detected between CD171-expression and MMR-deficiency or *EBER*-positivity (Supplementary Table S2).

**Table 5:** CD171 Expression in Colorectal Adenocarcinomas

Colorectal adenocarcinoma subtypes (n=210)	CD171 (tumor cells; %)
MMR-preserved (n=187)	64.2
MMR-deficient (n=23)	21.7**

Chi-square test

** p<0.01.

In oral cavity squamous carcinomas, those arisen in gingiva and tongue showed almost same CD171 positivity (46% and 47%, respectively), while those in tonsil showed much lower positivity (12%). The formers showed no statistical correlation between CD171- and p16-expression, whereas the latter showed significantly adverse correlation (Table 6).

**Table 6:** CD171 Expression in Oral Cavity SCCs

Oral cavity SCC subtypes	CD171 (tumor cells; %)
Tonsil (n=41)	
p16-negative (n=3)	66.7*
p16-positive (n=38)	7.9
Tongue (n=39)	
p16-negative (n=27)	51.9
p16-positive (n=12)	33.3
Gingiva (n=43)	
p16-negative (n=28)	46.4
p16-positive (n=15)	46.7

Fisher's exact test

* p<0.05.

SCC: squamous cell carcinoma

**Figure 4:**
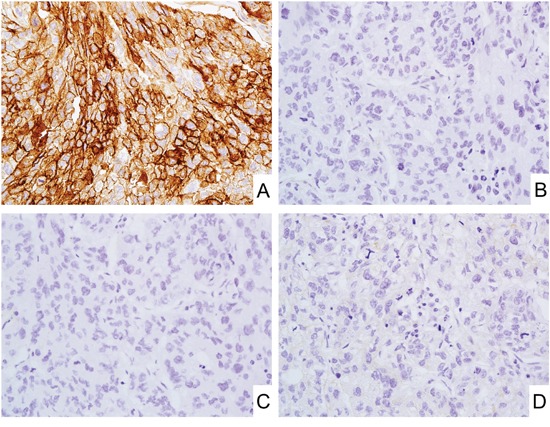
CD171 expression in triple-negative breast cancer **A-D.** Representative photos of the triple-negative breast cancer with strong and diffuse CD171 expression (A). ER (B), PgR (C), and human epidermal growth factor receptor (EGFR)-related 2 (HER2) (D) expressions were under-detectable level.

In breast cancer, subpopulations of ductal carcinomas showed weak to moderate CD171 expression (20%, median value of positive cells 40%). Two diffusely CD171-positive ductal carcinomas were triple-negative breast cancers (Figure 4A-4D). However, no CD171 expression was observed in lobular carcinoma.

In uterine endometrioid adenocarcinoma, 32 of 97 (33%) showed CD171 positivity in median 80% of tumor cells. As with breast cancer, we performed additional immunohistochemical staining for estrogen receptor (ER) and progesterone receptor (PgR), and this revealed 10 out of 32 cases showed partial or complete loss of the ER expression without PgR expression.

### Hematopoietic and lymphoid tumors

CD171 expression in lymphohematopoietic tumors has been summarized in Table 7. A minor population of diffuse large B-cell lymphoma (DLBCL, 7%), anaplastic large cell lymphoma (ALCL, 7%), and classical Hodgkin's lymphoma (4%) showed CD171 expression. In DLBCL and classical Hodgkin's lymphoma, no statistical correlation was detected between CD171- and *EBER*-expression (Supplementary Tables S3 and S4). There was also no statistical correlation between CD171- and ALK-expression in ALCLs (Supplementary Table S5).

**Table 7:** PD-L1 Expression in 310 Hematopoietic System Tumors

	Cases	/	All	(%)
Diffuse large B-cell lymphoma	6	/	84	7.1
Anaplastic large cell lymphoma	1	/	15	6.7
Classical Hodgkin's lymphoma	2	/	47	4.3
Follicular lymphoma	0	/	52	0.0
T-, B-Lymphoblastic lymphoma	0	/	8	0.0
Mantle cell lymphoma	0	/	33	0.0
Myeloid sarcoma	0	/	10	0.0
Nodal marginal zone lymphoma	0	/	6	0.0
Small cell lymphoma	0	/	19	0.0
Spleen, chronic myeloid leukemia	0	/	6	0.0
T-, NK-cell lymphoma	0	/	30	0.0

## DISCUSSION

CD171 was first identified in the CNS of the mouse as a neural cell adhesion molecule. [1] Studies indicated the importance of CD171 for normal development and maintenance of the nervous [10–12] and kidney tissue [14] as well as the immune system. [15] Subsequently, several reports showed CD171 overexpression lead to worse prognosis in various tumors [16–19] and there has been a growing interest in the treatment of CD171-expressing tumors by targeting this cell membrane molecule. [20–25] However, in several tumors, CD171 expression and correlation with prognosis remained controversial. [9, 26–30]

In this study, we evaluated the expression of CD171 using a newly developed rabbit monoclonal antibody offering an excellent signal quality with almost no background signals in normal tissues and 5155 tumors including germ cell, rare mesenchymal and lymphohematopoietic tumors to discuss the usefulness of CD171 immunohistochemistry in pathological diagnosis as well as pinpointing potential targets for future anti-CD171 therapy. We also analyzed here 1300 GISTs to asses previously reported conflicting immunohistochemical data. In addition, we performed survival analysis in conventional gastric GIST patients.

In normal adult tissue, CD171 was narrowly expressed in peripheral nerve bundles, brain, renal collecting ducts, vascular endothelial cells, and dendritic reticulum cells, as previously described (Supplementary Figure S1A-S1F). [14, 15, 27, 28]

In this study, only a small number of GISTs (1.5%, 20/1300) showed any CD171 expression, and most cases were only weakly positive (Figure 2C and 2D, Table 3). Moreover, there was no significant correlation between CD171 positivity and clinical outcome in conventional gastric GIST patients (Supplementary Figure S3). CD171-overexpression in GISTs was first reported using UJ127.11 antibody, with 73.6% (53/72) positivity. [31] Subsequently, several studies analyzed CD171 expression in 66-434 GIST cases and reported 55-59% of positivity with some studies suggesting the potential of CD171 expression for the prognostication of GIST. [29, 30, 32] Also, high serum concentration of the soluble CD171, metalloproteinase cleaved fragments of CD171, correlated with unfavorable outcome in GISTs patients in one study. [9] Whereas, different from past reports, CD171 does not appear to be useful for the GIST diagnosis or prognostication even when using the same mouse monoclonal antibodies as used in past reports. It is difficult to explain the difference in the frequency of CD171 positivity in GISTs. However, we consider our detection system proficient and had internal controls (peripheral nerve bundles) present in most cases (Supplementary Figure S2G-S2I).

Schwannomas, of the peripheral soft tissue and the gastrointestinal tract, showed typically homogeneous and strong cytoplasmic staining for CD171 (Figure 1A, Table 1). Neurofibromas showed weaker and sparser positivity for CD171 reflecting the heterogeneous composition of the tumor, however, their potential mimics such as benign fibrous histiocytoma, perineurioma, and solitary fibrous tumor were all negative for CD171 (Table 1). Therefore, this marker could be applied for their differential diagnosis.

True neural tumors such as neuroblastoma and paraganglioma are examples of strongly and consistently CD171-positive tumors, (Figure 1C and 1D) and in some cases, this marker could be useful for their diagnostic evaluation. Although one study reported better prognosis for CD171-positive neuroblastomas, our experience showed negative cases being so rare that CD171 is unlikely useful in the prognostication of neuroblastoma. [33]

Neuroendocrine tumors/carcinomas such as merkel cell carcinoma (Figure 1E), small cell carcinomas of the lung (Figure 1F), and pancreatic neuroendocrine tumors were also typically positive for CD171 suggesting a potential application of CD171 immunohistochemistry in their diagnosis.

In other epithelial and mesenchymal tumor(s), significant CD171 expression was observed in ovary serous carcinoma (87%, Figure 3A), malignant mesothelioma (70%, Figure 3B), testicular embryonal carcinoma (60%), colorectal adenocarcinoma (56%), and alveolar rhabdomyosarcoma (50%, Figure 2A). Among them, within our best knowledge, its frequent expressions in alveolar rhabdomyosarcoma and embryonal carcinoma were first documented in this report. In addition, negative correlations between CD171-expression and MMR-deficiency or p16-expression in colorectal adenocarcinoma and tonsil squamous cell carcinoma also have not been documented (Table 5 and 6).

In hormone-dependent female cancers such as breast and endometrial cancers, CD171 expression has a negative correlation with ER/PgR expression in both breast and endometrial carcinomas in agreement with previous reports. [34–36] The rarity of CD171 expression in prostate cancer (<2%) could be related to its reported down-regulation by androgen receptor. [34] Recent reports also showed that several miRNAs (miR-34a) and transcription factors (β-catenin and slug) regulate CD171 expression in endometrial adenocarcinomas. [37, 38] The CD171 regulatory mechanisms in other tumors should be elucidated in the near future.

At first, several types of anti-CD171 monoclonal antibodies were introduced to the imaging and radioimunotherapeutic targeting of neuroblastoma in xenograft models. [21, 23] Subsequently, other CD171-expressing tumors such as ovarian cancer, malignant melanoma and pancreatic adenocarcinoma were also targeted by anti-CD171 antibodies and indeed, some of them showed anti-tumor effects without significant side effects. [20, 25] Recently, chimeric antigen receptor redirected T lymphocytes were established and also introduced to the treatment of neuroblastoma and other malignancies. [22, 24] Thus, there has been a growing interest in targeting this cell membrane molecule on neoplastic cells. In this study, we would like to propose newly identified CD171-highly expressing neoplasms such as alveolar rhabdomyosarcoma and embryonal carcinoma as well as MMR-preserved colorectal adenocarcinomas and p16-negative tonsil squamous cell carcinoma as potential targets for future CD171-targeting therapy.

In conclusion, CD171 immunohistochemistry with a new rabbit monoclonal antibody may assist diagnosis of neural or neuroectodermal tumors such as schwannoma, neuroblastoma, and, paraganglioma due to their consistent positivity. CD171 could also be a useful marker for the other neuroendocrine tumors while its expression in non-neuroendocrine tumors has to be taken into account. We would like to propose these CD171-highly expressing neoplasms as potential targets for anti-CD171 therapy. However, CD171 is not useful for GIST diagnosis or prognostication as previously proposed, based on its rare expression and lack of correlation with outcome.

## MATERIALS AND METHODS

5155 anonymized tumors including epithelial, mesenchymal, melanocytic, and lymphohematopoietic tumors were collected. This project was completed under Office of Human Subject Research Exemption with anonymized specimens. Immunohistochemistry was performed on sections of multitumor blocks containing 30 to 60 rectangular tissue samples, assembled and embedded in paraffin as previously described. [39] The size of tumor tissue samples was estimated to exceed the size of a single 0.6 mm^2^ core by a factor of 10-15. All tumors, selected for this study, were extensively documented histologically and immunohistochemically. Also, a panel of normal tissues including adrenocortical gland, brain, gastrointestinal- and genitourinary-tract, kidney, liver, lung, pancreas, placenta, thyroid gland and lymphatic tissue was evaluated for CD171 expression.

The primary rabbit monoclonal antibody clone 014 against CD171 (10140-R014) was obtained from Sino Biological Inc. (Beijing, China) and the primary mouse monoclonal antibodies clone UJ127.11 and clone L1-14.10 were from Thermo Fisher Scientific (Waltham, MA, USA) and Biolegend (San Diego, CA, USA), respectively. For immunohistochemical staining, rabbit monoclonal antibody was used at a dilution of 1:500. Both UJ127.11 and L1-14.10 mouse monoclonal antibodies were used at a dilution of 1:150. Immunostaining was performed using the Leica Bond-Max automation and Leica Refine detection kit (Leica Biosystems, Bannockburn, IL). The approximately 3-hour protocol included in situ deparaffinization and high-pH epitope retrieval for 25 minutes and incubation with primary antibody for 30 minutes, polymer for 15 minutes, postpolymer for 15 minutes, and DAB as the chromogen for 10 minutes, followed by 5-minute hematoxylin counterstaining. MutL Homolog 1 (MLH1), MutS Homolog 2 (MSH2), MutS Homolog 6 (MSH6), and PMS2 immunohistochemistry was performed to analyze MMR system status as previously reported. [40] For the detection of Epstein-Barr virus (EBV) infection, Bond− Ready-to-Use ISH EBER Probe was used in Leica Bond-Max automation system according to the manufacturer instructions (Leica Biosystems, Bannockburn, IL).

CD171 immunoreactivity with peripheral nerves was used as an internal positive control. The stained sections were independently evaluated by two pathologists (SI and MM).

Chi-square test or Fisher's exact test were performed by SPSS software (IBM, Armonk, NY) to analyze the statistical correlation between CD171-expression and other tumor status such as MMR-deficiency, *EBER*-, p16-, and ALK-expression. For the survival analysis of GIST patients, EZR version 1.32 was used. [41] We performed survival analysis only in conventional gastric GIST patients because of insufficient patient numbers with CD171-positive GIST of other sites.

## SUPPLEMENTARY FIGURES AND TABLES



## References

[1] Rathjen FG, Schachner M (1984). Immunocytological and biochemical characterization of a new neuronal cell surface component (L1 antigen) which is involved in cell adhesion. The EMBO journal.

[2] Moos M, Tacke R, Scherer H, Teplow D, Fruh K, Schachner M (1988). Neural adhesion molecule L1 as a member of the immunoglobulin superfamily with binding domains similar to fibronectin. Nature.

[3] Maness PF, Schachner M (2007). Neural recognition molecules of the immunoglobulin superfamily: signaling transducers of axon guidance and neuronal migration. Nature neuroscience.

[4] Schachner M (1997). Neural recognition molecules and synaptic plasticity. Current opinion in cell biology.

[5] Brummendorf T, Kenwrick S, Rathjen FG (1998). Neural cell recognition molecule L1: from cell biology to human hereditary brain malformations. Current opinion in neurobiology.

[6] Herron LR, Hill M, Davey F, Gunn-Moore FJ (2009). The intracellular interactions of the L1 family of cell adhesion molecules. The Biochemical journal.

[7] Beer S, Oleszewski M, Gutwein P, Geiger C, Altevogt P (1999). Metalloproteinase-mediated release of the ectodomain of L1 adhesion molecule. Journal of cell science.

[8] Gutwein P, Oleszewski M, Mechtersheimer S, Agmon-Levin N, Krauss K, Altevogt P (2000). Role of Src kinases in the ADAM-mediated release of L1 adhesion molecule from human tumor cells. The Journal of biological chemistry.

[9] Zander H, Rawnaq T, von Wedemeyer M, Tachezy M, Kunkel M, Wolters G, Bockhorn M, Schachner M, Izbicki JR, Kaifi J (2011). Circulating levels of cell adhesion molecule L1 as a prognostic marker in gastrointestinal stromal tumor patients. BMC cancer.

[10] Lindner J, Rathjen FG, Schachner M (1983). L1 mono- and polyclonal antibodies modify cell migration in early postnatal mouse cerebellum. Nature.

[11] Martini R, Schachner M (1986). Immunoelectron microscopic localization of neural cell adhesion molecules (L1, N-CAM, and MAG) and their shared carbohydrate epitope and myelin basic protein in developing sciatic nerve. The Journal of cell biology.

[12] Cohen NR, Taylor JS, Scott LB, Guillery RW, Soriano P, Furley AJ (1998). Errors in corticospinal axon guidance in mice lacking the neural cell adhesion molecule L1. Current biology: CB.

[13] Fransen E, Van Camp G, Vits L, Willems PJ (1997). L1-associated diseases: clinical geneticists divide, molecular geneticists unite. Human molecular genetics.

[14] Debiec H, Christensen EI, Ronco PM (1998). The cell adhesion molecule L1 is developmentally regulated in the renal epithelium and is involved in kidney branching morphogenesis. The Journal of cell biology.

[15] Pancook JD, Reisfeld RA, Varki N, Vitiello A, Fox RI, Montgomery AM (1997). Expression and regulation of the neural cell adhesion molecule L1 on human cells of myelomonocytic and lymphoid origin. J Immunol.

[16] Kajiwara Y, Ueno H, Hashiguchi Y, Shinto E, Shimazaki H, Mochizuki H, Hase K (2011). Expression of l1 cell adhesion molecule and morphologic features at the invasive front of colorectal cancer. American journal of clinical pathology.

[17] Tischler V, Pfeifer M, Hausladen S, Schirmer U, Bonde AK, Kristiansen G, Sos ML, Weder W, Moch H, Altevogt P, Soltermann A (2011). L1CAM protein expression is associated with poor prognosis in non-small cell lung cancer. Molecular cancer.

[18] Zecchini S, Bianchi M, Colombo N, Fasani R, Goisis G, Casadio C, Viale G, Liu J, Herlyn M, Godwin AK, Nuciforo PG, Cavallaro U (2008). The differential role of L1 in ovarian carcinoma and normal ovarian surface epithelium. Cancer research.

[19] Zeimet AG, Reimer D, Huszar M, Winterhoff B, Puistola U, Azim SA, Muller-Holzner E, Ben-Arie A, van Kempen LC, Petru E, Jahn S, Geels YP, Massuger LF, Amant F, Polterauer S, Lappi-Blanco E (2013). L1CAM in early-stage type I endometrial cancer: results of a large multicenter evaluation. Journal of the National Cancer Institute.

[20] Doberstein K, Harter PN, Haberkorn U, Bretz NP, Arnold B, Carretero R, Moldenhauer G, Mittelbronn M, Altevogt P (2015). Antibody therapy to human L1CAM in a transgenic mouse model blocks local tumor growth but induces EMT. International journal of cancer.

[21] Hoefnagel CA, Rutgers M, Buitenhuis CK, Smets LA, de Kraker J, Meli M, Carrel F, Amstutz H, Schubiger PA, Novak-Hofer I (2001). A comparison of targeting of neuroblastoma with mIBG and anti L1-CAM antibody mAb chCE7: therapeutic efficacy in a neuroblastoma xenograft model and imaging of neuroblastoma patients. European journal of nuclear medicine.

[22] Hong H, Stastny M, Brown C, Chang WC, Ostberg JR, Forman SJ, Jensen MC (2014). Diverse solid tumors expressing a restricted epitope of L1-CAM can be targeted by chimeric antigen receptor redirected T lymphocytes. J Immunother.

[23] Novak-Hofer I, Amstutz HP, Haldemann A, Blaser K, Morgenthaler JJ, Blauenstein P, Schubiger PA (1992). Radioimmunolocalization of neuroblastoma xenografts with chimeric antibody chCE7. Journal of nuclear medicine.

[24] Park JR, Digiusto DL, Slovak M, Wright C, Naranjo A, Wagner J, Meechoovet HB, Bautista C, Chang WC, Ostberg JR, Jensen MC (2007). Adoptive transfer of chimeric antigen receptor re-directed cytolytic T lymphocyte clones in patients with neuroblastoma. Molecular therapy.

[25] Schafer H, Dieckmann C, Korniienko O, Moldenhauer G, Kiefel H, Salnikov A, Kruger A, Altevogt P, Sebens S (2012). Combined treatment of L1CAM antibodies and cytostatic drugs improve the therapeutic response of pancreatic and ovarian carcinoma. Cancer letters.

[26] Allory Y, Matsuoka Y, Bazille C, Christensen EI, Ronco P, Debiec H (2005). The L1 cell adhesion molecule is induced in renal cancer cells and correlates with metastasis in clear cell carcinomas. Clinical cancer research.

[27] Doberstein K, Wieland A, Lee SB, Blaheta RA, Wedel S, Moch H, Schraml P, Pfeilschifter J, Kristiansen G, Gutwein P (2011). L1-CAM expression in ccRCC correlates with shorter patients survival times and confers chemoresistance in renal cell carcinoma cells. Carcinogenesis.

[28] Huszar M, Moldenhauer G, Gschwend V, Ben-Arie A, Altevogt P, Fogel M (2006). Expression profile analysis in multiple human tumors identifies L1 (CD171) as a molecular marker for differential diagnosis and targeted therapy. Human pathology.

[29] Du Y, Zhang H, Jiang Z, Huang G, Lu W, Wang H (2015). Expression of L1 protein correlates with cluster of differentiation 24 and integrin beta1 expression in gastrointestinal stromal tumors. Oncology letters.

[30] Steigen SE, Bjerkehagen B, Haugland HK, Nordrum IS, Loberg EM, Isaksen V, Eide TJ, Nielsen TO (2008). Diagnostic and prognostic markers for gastrointestinal stromal tumors in Norway. Modern pathology.

[31] Kaifi JT, Strelow A, Schurr PG, Reichelt U, Yekebas EF, Wachowiak R, Quaas A, Strate T, Schaefer H, Sauter G, Schachner M, Izbicki JR (2006). L1 (CD171) is highly expressed in gastrointestinal stromal tumors. Modern pathology.

[32] Rawnaq T, Quaas A, Zander H, Gros SJ, Reichelt U, Blessmann M, Wilzcak W, Schachner M, Sauter G, Izbicki JR, Kaifi JT (2012). L1 is highly expressed in tumors of the nervous system: a study of over 8000 human tissues. The Journal of surgical research.

[33] Wachowiak R, Fiegel HC, Kaifi JT, Quaas A, Krickhahn A, Schurr PG, Erttmann R, Schachner M, Kluth D, Sauter G, Izbicki JR (2007). L1 is associated with favorable outcome in neuroblastomas in contrast to adult tumors. Annals of surgical oncology.

[34] Doberstein K, Milde-Langosch K, Bretz NP, Schirmer U, Harari A, Witzel I, Ben-Arie A, Hubalek M, Muller-Holzner E, Reinold S, Zeimet AG, Altevogt P, Fogel M (2014). L1CAM is expressed in triple-negative breast cancers and is inversely correlated with androgen receptor. BMC cancer.

[35] Huszar M, Pfeifer M, Schirmer U, Kiefel H, Konecny GE, Ben-Arie A, Edler L, Munch M, Muller-Holzner E, Jerabek-Klestil S, Abdel-Azim S, Marth C, Zeimet AG, Altevogt P, Fogel M (2010). Up-regulation of L1CAM is linked to loss of hormone receptors and E-cadherin in aggressive subtypes of endometrial carcinomas. The Journal of pathology.

[36] Schroder C, Schumacher U, Fogel M, Feuerhake F, Muller V, Wirtz RM, Altevogt P, Krenkel S, Janicke F, Milde-Langosch K (2009). Expression and prognostic value of L1-CAM in breast cancer. Oncology reports.

[37] Pfeifer M, Schirmer U, Geismann C, Schafer H, Sebens S, Altevogt P (2010). L1CAM expression in endometrial carcinomas is regulated by usage of two different promoter regions. BMC molecular biology.

[38] Schirmer U, Doberstein K, Rupp AK, Bretz NP, Wuttig D, Kiefel H, Breunig C, Fiegl H, Muller-Holzner E, Zeillinger R, Schuster E, Zeimet AG, Sultmann H, Altevogt P (2014). Role of miR-34a as a suppressor of L1CAM in endometrial carcinoma. Oncotarget.

[39] Miettinen M (2012). A simple method for generating multitissue blocks without special equipment. Applied immunohistochemistry & molecular morphology.

[40] Lasota J, Kowalik A, Wasag B, Wang ZF, Felisiak-Golabek A, Coates T, Kopczynski J, Gozdz S, Miettinen M (2014). Detection of the BRAF V600E mutation in colon carcinoma: critical evaluation of the imunohistochemical approach. The American journal of surgical pathology.

[41] Kanda Y (2013). Investigation of the freely available easy-to-use software ‘EZR’ for medical statistics. Bone marrow transplantation.

