# Supplementary material for: Expression of neural cell adhesion molecule L1 (CD171) in neuroectodermal and other tumors. An immunohistochemical study of 5155 tumors and critical evaluation of CD171 prognostic value in gastrointestinal stromal tumors
Source: Oncotarget. 2016 Jul 11;7(34):55276–89. doi: 10.18632/oncotarget.10527 (PMC5338914; doi:10.18632/oncotarget.10527)
Supplement: Supplementary file 1 [file oncotarget-07-55276-s001.pdf]

Expression of neural cell adhesion molecule L1 (CD171) in neuroectodermal and other tumors. An immunohistochemical study of 5155 tumors and critical evaluation of CD171 prognostic value in gastrointestinal stromal tumors

SUPPLEMENTARY FIGURES AND TABLES

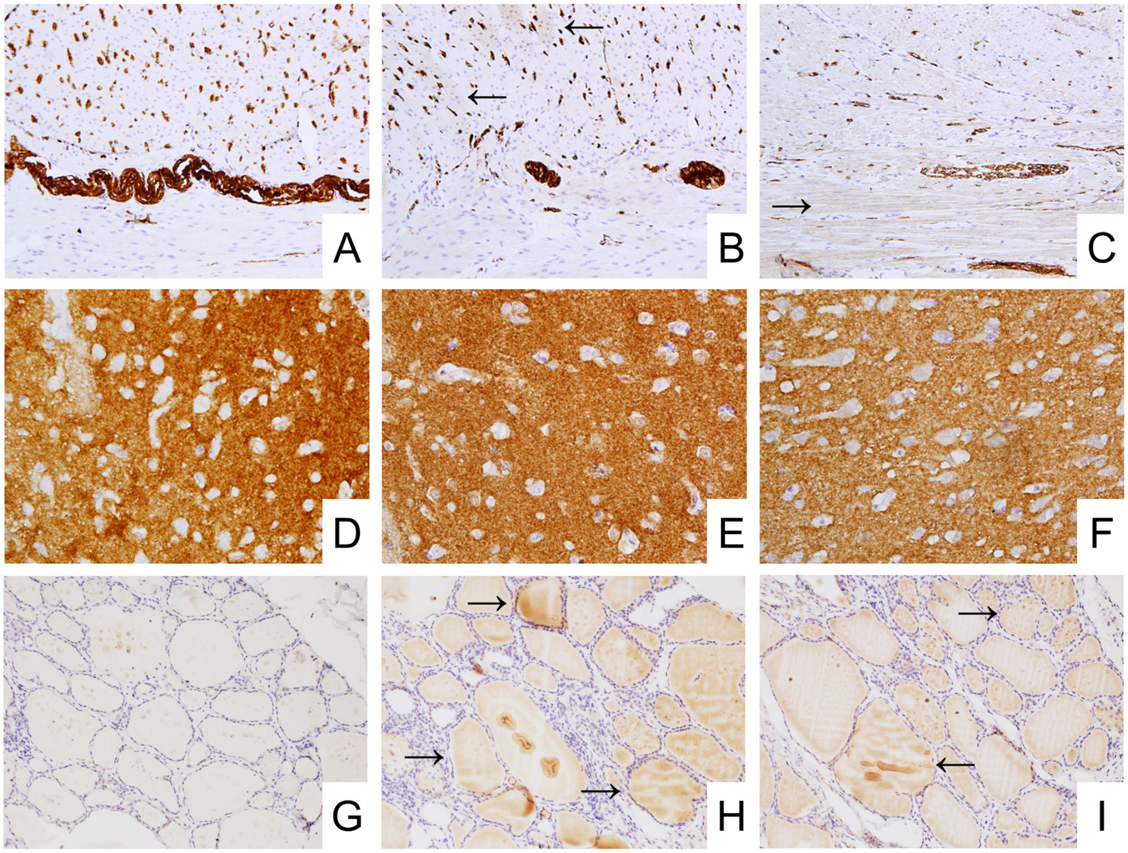

Supplementary Figure S1: CD171 immunohistochemical staining in normal tissues using three different monoclonal antibodies. A-C. CD171 was strongly expressed in nerve bundles located within smooth muscle layer of the stomach. **D-F.** Cerebrum showed strong CD171 expression. **G-I.** CD171 immunostaining of thyroid glands. A, D, and G were stained by rabbit monoclonal antibody. B, E, and H were stained by mouse monoclonal antibody L1-14.10. C, F, and I were stained by mouse monoclonal antibody UJ127.11. Note that rabbit monoclonal antibody showed weakest background signals. Whereas, mouse monoclonal antibodies L1-14.10 and UJ127.11 showed higher background signals (arrow).

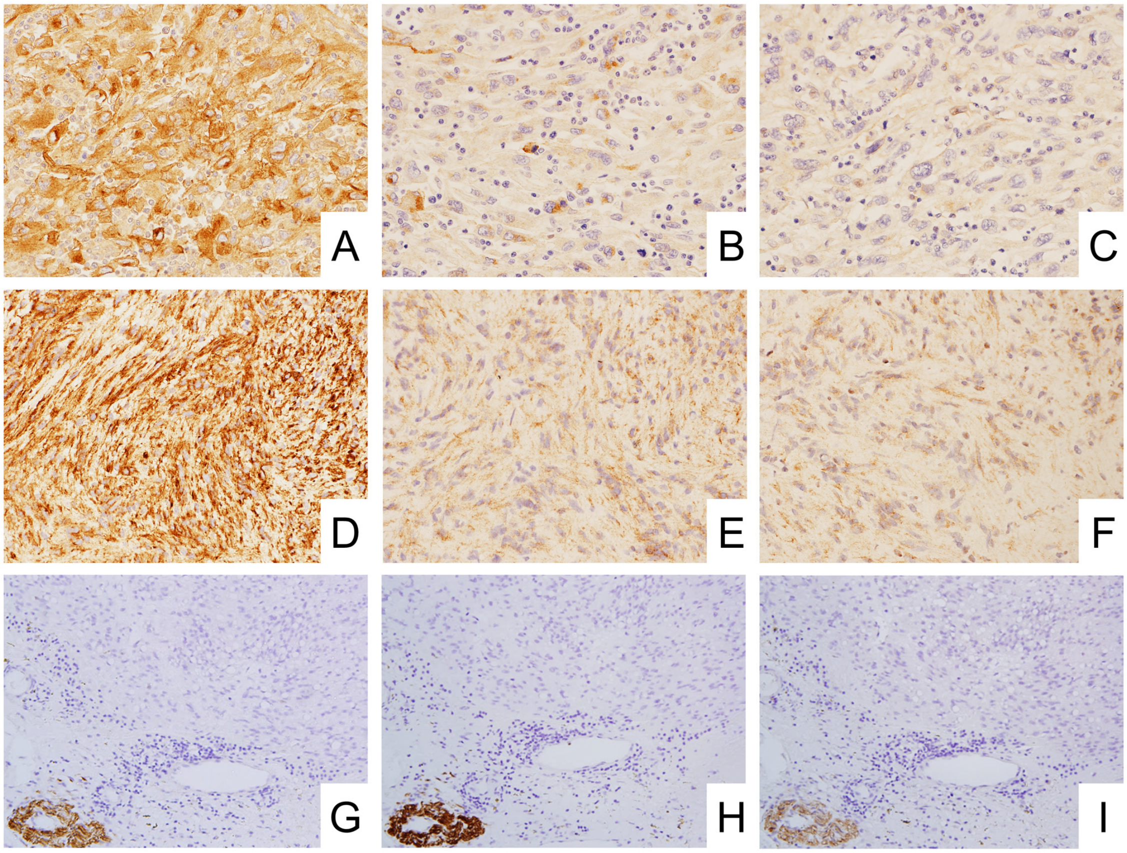

Supplementary Figure S2: CD171 immunohistochemical staining in clear cell renal carcinoma, schwannoma, and conventional gastric GIST using three different monoclonal antibodies. A-C. CD171 immunostaining of a case of clear cell renal cell carcinoma with sarcomatoid feature. **D-F.** CD171 immunostaining of a case of schwannoma. **G-I.** CD171 expression was not detected in majority of GISTs except for the nervous bundles around the tumor foci. A, D, and G were stained by rabbit monoclonal antibody 014. B, E, and H were stained by mouse monoclonal antibody L1-14.10. C, F, and I were stained by mouse monoclonal antibody UJ127.11. Note that rabbit monoclonal antibody showed stronger signals than others in 3 cases of clear cell renal cell carcinoma and a case of schwannoma. In a few GIST cases, mouse monoclonal antibody L1-14.10 showed stronger signal than others in the normal peripheral nerve bundles.

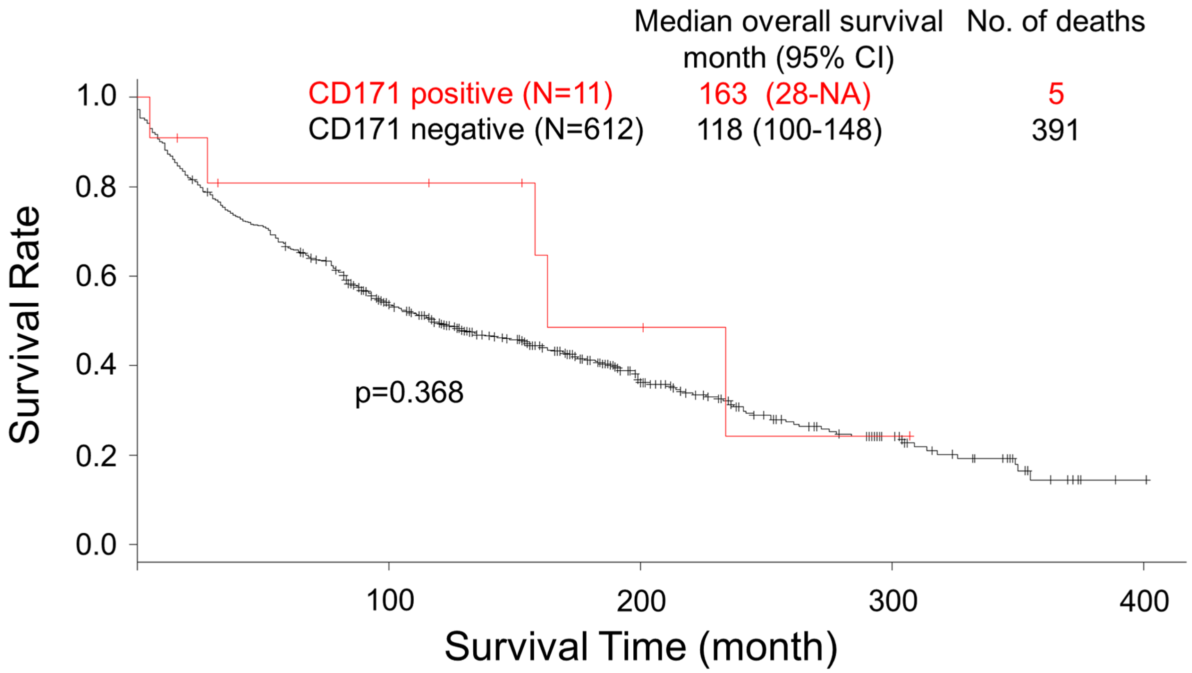

Supplementary Figure S3: Overall survival for patients expressing CD171 (red line) compared with patients not expressing CD171 (black line). No significant correlation was identified between the expression of CD171 and patient survival.

Supplementary Table S1: Comparison of Three Different Monoclonal Antibodies against CD171

	Clone 014		Clone L1-14.10		Clone UJ127.11	
	**case**	**(%)**	**case**	**(%)**	**case**	**(%)**
Clear cell renal cell carcinoma (n=42)						
Negative	30	(71.4)	33	(78.6)	34	(81.0)
Weak	2	(4.8)	2	(4.8)	3	(7.1)
Strong	10	(23.8)	7	(16.7)	5	(11.9)
Schwannoma (n=56)						
Negative	1	(1.8)	1	(1.8)	2	(3.6)
Weak	1	(1.8)	2	(3.6)	4	(7.1)
Strong	54	(96.4)	53	(94.6)	50	(89.3)
Stomach, conventional GIST (n=57)						
Negative	56	(98.2)	57	(100)	57	(100)
Weak	1	(1.8)	0	(0.0)	0	(0.0)
Strong	0	(0.0)	0	(0.0)	0	(0.0)
Pancreas, NETs (n=42)						
Negative	11	(26.2)	11	(26.2)	20	(47.6)
Weak	21	(50.0)	21	(50.0)	18	(42.9)
Strong	10	(23.8)	10	(23.8)	4	(9.5)
Paraganglioma (n=43)						
Negative	3	(7.0)	3	(7.0)	16	(37.2)
Weak	1	(2.3)	1	(2.3)	7	(16.3)
Strong	39	(90.7)	39	(90.7)	20	(46.5)

The staining intensities were semi-quantitatively scored using a three-tiered scale (negative, weak, and strong). NETs: neuroendocrine tumors.

Supplementary Table S2: CD171 Expression in Gastric Adenocarcinomas

**Gastric adenocarcinoma subtypes (n=97 in MMR analysis; n=100 in *EBER* analysis)**	**CD171 (tumor cells; %)**
MMR-preserved (n=92)	31.5
MMR-deficient (n=5)	20.0
EBER-negative (n=90)	33.3
EBER-positive (n=10)	10.0

Fisher's exact test.

Supplementary Table S3: CD171 Expression in DLBCLs

**DLBCL subtypes (n=84)**	**CD171 (tumor cells; %)**
EBER-negative (n=82)	7.9
EBER-positive (n=2)	0.0

Fisher's exact test
DLBCL: Diffuse large B-cell lymphoma

Supplementary Table S4: CD171 Expression in Classical Hodgkin's Lymphomas

Classical Hodgkin's lymphoma subtypes (n=47)	CD171 (tumor cells; %)
EBER-negative (n=33)	0.0
EBER-positive (n=14)	14.3

Fisher's exact test

Supplementary Table S5: CD171 Expression in ALCLs

ALCL subtypes (n=15)	PD-L1 (tumor cells; %)
ALK-negative (n=10)	10.0
ALK-positive (n=5)	0.0

Fisher's exact test
ALCL: anaplastic large cell lymphoma
